# A Niche for Infectious Disease in Environmental Health: Rethinking the Toxicological Paradigm

**DOI:** 10.1289/ehp.0901866

**Published:** 2010-04-12

**Authors:** Beth J. Feingold, Leora Vegosen, Meghan Davis, Jessica Leibler, Amy Peterson, Ellen K. Silbergeld

**Affiliations:** 1 Department of Environmental Health Sciences and; 2 Department of Epidemiology, Johns Hopkins Bloomberg School of Public Health, Baltimore, Maryland, USA

**Keywords:** biomarkers, colon cancer, conceptual framework, environmental health, infectious disease, liver cancer, NIEHS, pathogens, toxicology

## Abstract

**Objective:**

In this review we highlight the need to expand the scope of environmental health research, which now focuses largely on the study of toxicants, to incorporate infectious agents. We provide evidence that environmental health research would be strengthened through finding common ground with the tools and approaches of infectious disease research.

**Data sources and extraction:**

We conducted a literature review for examples of interactions between toxic agents and infectious diseases, as well as the role of these interactions as risk factors in classic “environmental” diseases. We investigated existing funding sources and research mandates in the United States from the National Science Foundation and the National Institutes of Health, particularly the National Institute of Environmental Health Sciences.

**Data synthesis:**

We adapted the toxicological paradigm to guide reintegration of infectious disease into environmental health research and to identify common ground between these two fields as well as opportunities for improving public health through interdisciplinary research.

**Conclusions:**

Environmental health encompasses complex disease processes, many of which involve interactions among multiple risk factors, including toxicant exposures, pathogens, and susceptibility. Funding and program mandates for environmental health studies should be expanded to include pathogens in order to capture the true scope of these overlapping risks, thus creating more effective research investments with greater relevance to the complexity of real-world exposures and multifactorial health outcomes. We propose a new model that integrates the toxicology and infectious disease paradigms to facilitate improved collaboration and communication by providing a framework for interdisciplinary research. Pathogens should be part of environmental health research planning and funding allocation, as well as applications such as surveillance and policy development.

Environmental health and infectious disease have been intertwined in the study of public health since at least the mid-1850s, when John Snow used an environmental map to determine origins of a London cholera outbreak, thus highlighting the concept of place as a determinant of infectious disease prevalence and transmission risks. Since that time, the fields of environmental health and infectious disease have diverged in many regions, particularly in the United States, where these fields are currently treated as distinct entities, leading to separate research funding tracks and distinct training programs in schools of public health. Although environmental health research continues to contribute to understanding key factors relevant to infectious diseases such as malaria, Lyme disease, and hantavirus pulmonary syndrome (Peterson et al., in press), environmental health research and practice predominantly focus on chemical and physical agents despite the inherent role of the environment in pathogen dynamics and host response.

Environmental health’s focus on the abiotic factors of toxicology has meant that the field produces relatively little research on how pathogens and toxic agents interact to increase risks and severity of diseases and dysfunctions. Several of the major toxicant exposures studied within environmental health research alter risks for pathogen transmission and disease severity. Exposures to toxic agents may lead to immunotoxic changes in hosts that reduce the threshold for infection, increase the persistence of an infection, increase pathogen shedding, and alter the severity and burden of infectious disease. Pathogens can modify inflammatory pathways and other responses induced by environmental toxicants and modify the likelihood and severity of chronic disease progression. For these reasons, in this article we argue that infectious diseases should be considered explicitly within the toxicological framework to capture interactions between pathogens and toxicants that contribute to the etiology of diseases often assumed to be of either pathogen or toxicant origin.

In this article, specific examples highlight the importance of unifying infectious disease and toxicant research. In particular, interactions between a toxic agent (aflatoxin) and a pathogen [hepatitis B virus (HBV)] dramatically increase the risk of liver cancer (Groopman et al. 2005). However, researchers have not designed studies specifically to address potential interactions in the etiology of other chronic diseases, such as the potential interaction of toxicants (Rohrmann et al. 2009) and microbial agents in colon cancer etiology. Environmental agents can be important cofactors even in diseases for which a pathogen plays a primary role, as in cervical cancer, in which human papilloma virus (HPV) is the primary etiological factor but smoking may act as a cofactor (Castellsagué and Muñoz 2003). These examples illustrate the benefits of reincorporating infectious disease research into environmental health.

## Background

### Historical context

The relatively recent distancing between research and public health practice in the fields of infectious disease and environmental health stems in large part from the influence of the conservation movement on the development of environmental health in the United States, as well as on shifting political priorities in the late 20th century (McNeill 2000). Before the establishment of environmental agencies in the 1970s, the U.S. Public Health Service ran environmental health programs that focused on identification and prevention of infectious disease through the disciplines of sanitation, microbiology, and pathobiology. The establishment of the U.S. Environmental Protection Agency (EPA) launched a distinct environmental health agenda that largely excluded infectious disease research and control. The U.S. EPA inherited the authority to regulate air and water, but the Public Health Service kept infectious disease control. Other countries have replicated this pattern, excluding infectious disease research and practice from environmental health; for example, Brazilian, German, Swedish, and Japanese environmental agencies focus exclusively on chemical risks and toxicological research. The present structure of the World Health Organization (WHO) echoes this divide between environmental and infectious disease programs. This programmatic separation has been problematic, exemplified in the conflict within the WHO over the revived use of DDT (dichlorodiphenyltrichloroethane) for malaria control in 2006 (Eskenazi et al. 2009; van den Berg 2009).

Even within Public Health Service agencies concerned with the environment, such as the National Institute of Environmental Health Sciences (NIEHS), research has not emphasized infectious disease. Competing niches in research, and the much earlier establishment of the National Institute of Allergy and Infectious Diseases (NIAID), may have contributed to this. Through the 1970s, political decisions (e.g., the creation of the NIEHS National Toxicology Program and the U.S. EPA Superfund Program) formalized close interactions between the research agenda of the NIEHS and the regulatory program of the U.S. EPA, further contributing to the NIEHS research agenda on toxicology and the exclusion of infectious disease research. The NIEHS expanded its purview into global environmental health in 2006, but none of the extramurally funded projects under this initiative have focused on infectious disease per se (Drew et al. 2008).

Over the past 20 years, the NIEHS and NIAID have diverged in intramural and extramural research priorities (NIAID 2009). Although promising, the Ecology of Infectious Diseases Initiative (EID) coordinated by the National Science Foundation and the Fogarty International Center has failed to bridge the research gap between toxicology and infectious disease. According to its stated goal, the EID (National Science Foundation 2010) supports

the development of predictive models and the discovery of principles governing the transmission dynamics of infectious disease agents. To that end, research proposals should focus on understanding the ecological and socio-ecological determinants of transmission by vectors or abiotic agents, the population dynamics of reservoir species, the transmission to humans or other hosts, or the cultural, social, behavioral, and economic dimensions of disease communication.

However, no projects funded by the EID to date have studied interactions between toxicant exposures and infectious disease.

### NIEHS funding priorities and research agenda

The stated mission of the NIEHS (2009) is to “reduce the burden of human illness and disability by understanding how the environment influences the development and progression of human disease.” This has led to a concentration on basic science and training related to disease-oriented research and global environmental health. Intramural research at the NIEHS includes five programs: environmental biology, environmental disease and medicine, clinical research, environmental toxicology, and the National Toxicology Program. Extramurally, the top five funded areas of organ systems or disease research are respiratory, neurodegenerative and neurodevelopmental, endocrine (including breast cancer), cardiovascular, and immune system diseases or disorders. Basic science research funding focuses on laboratory-based inquiry more than on disease processes in the context of the environment or large populations.

NIEHS training grants support research that aims to “understand how environmental exposures alter biologic processes and affect the risk of either disease development or the distribution of disease in populations” (NIEHS 2006). These training grants aim to support research not only on chemicals known to be environmental pollutants but also on “fungal or bacterially derived toxins due to ambient exposures” as well as those infectious and parasitic agents that are “disease co-factors with an environmental toxicant exposure” (NIEHS 2006). Although the scope of the award includes this language, most funded projects to date do not encompass these areas.

### Problem formulation

Pathogens are not included in the current toxicological paradigm, despite their contribution to the etiology of environmental disease. Therefore, we developed a toxicological–pathogen conceptual paradigm as a framework for integrating toxicology and infectious disease research and theory.

## The Integrated Conceptual Paradigm for Environmental Disease Etiology

**Figure 1:**
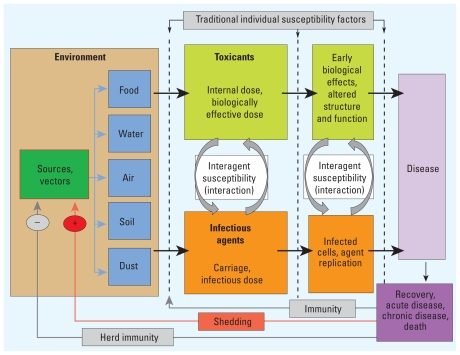
Integrated toxicological–pathogen conceptual paradigm for disease etiology.

**Figure 2:**
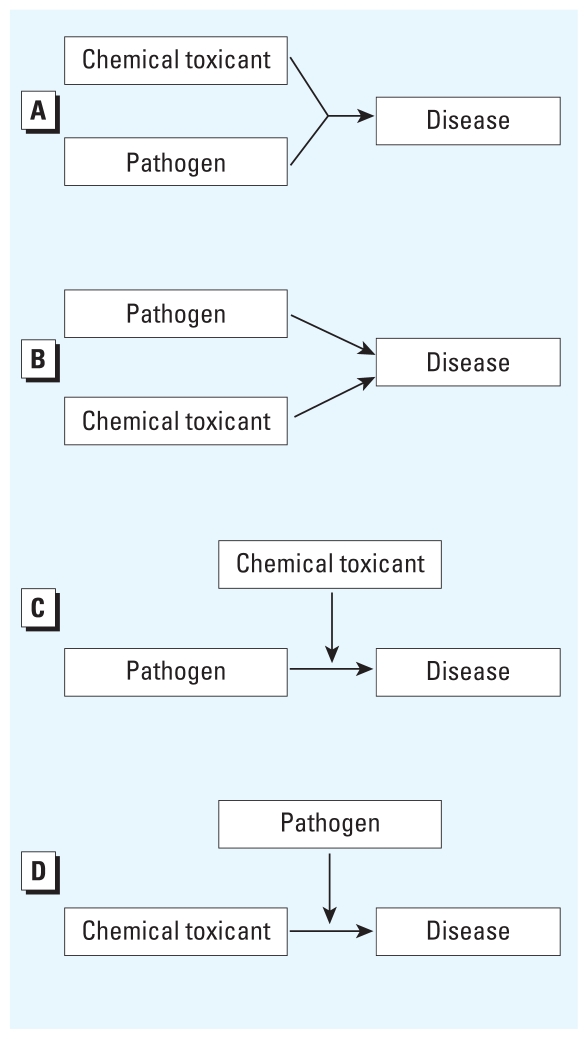
Four potential scenarios showing potential causality and interactions between pathogens and toxicants in disease etiology. (*A*) A toxicant and a pathogen may both be required to act together to cause a disease. (*B*) Either a pathogen or a toxicant alone is sufficient to cause the disease. (*C*) A chemical toxicant can modify the association between a pathogen and a disease. (*D*) A pathogen can modify the association between a chemical toxicant and a disease. Adapted from Ottman (1996).

Figure 1 presents our integrated paradigm for environmental health, combining the toxicological paradigm (National Research Council Committee on Biological Markers 1987) with aspects of conceptual models of infectious disease transmission (Anderson and May 1992). Our integrated model specifically incorporates potential interactions, in contrast to existing toxicological and infectious disease models that assume one agent causes one disease. In reality, disease etiology is more complex, and in Figure 2, we adapt the Ottman model (Ottman 1996) for gene–environment interactions to demonstrate how two risk factors, specifically toxicants and pathogens, can interact to cause disease. Our integrated paradigm (Figure 1) captures these possibilities by including interagent interactions at several stages along the backbone of the toxicological paradigm, which models progression from an individual’s exposure to clinical disease.

Some differences between the toxicological paradigm and the infectious disease model challenge their integration. Although some epidemiological studies in environmental health focus on populations [largely with ecological designs, e.g., those in air pollution research (Bell et al. 2009; Katsouyanni et al. 2009)], the classic toxicological paradigm models toxicokinetics and toxicodynamics at the level of the individual, generalizing from this level to a similarly exposed population. In contrast, models of infection are based on population-level probabilities of progressing from exposure to infected, infectious, or immune/susceptible states. The concept of “risk transmission” is critical to infectious disease dynamics. In contrast, environmental toxicant transfers generally do not occur through direct person-to-person transmission. However, fomite transmission does occur for both infectious and toxic agents; pathogens may be transmitted via inanimate objects, and similarly, workers may transport toxicants to their homes on vehicles or clothing (McDiarmid and Weaver 1993). Interindividual transmission of both infectious and toxic agents can occur through mother-to-child transmission, as in the case of HIV (human immunodeficiency virus) and the toxicants bisphenol A and mercury (Barragan and Sibley 2003; Sakamoto et al. 2010; Schonfelder et al. 2002).

In infectious disease modeling, the additional consideration of change in host susceptibility (or immune status) that occurs in the context of infection is critical. In contrast, toxicologists generally do not consider exposure itself to modulate susceptibility, instead defining susceptibility factors as preexisting independent of exposure, such as genetic factors (National Research Council Committee on Biological Markers 1987). In some cases, enzyme induction may reduce or increase susceptibility on a transient basis by enhancing detoxification or activation, but this is usually a matter of degree and not absolute resistance in the immunological sense. No evidence exists that prior exposures to toxic agents prevent intoxication from later exposures; rather, cumulative exposures to most toxicants increase risks of adverse health outcomes.

Another challenge is that in toxicological models the quantity of an agent does not change over short periods of time in the environment, whereas infectious disease models must incorporate dynamics of growth or die-off of microbial populations. Pathogens are unique in that they can evolve such that the nature of their hazard changes (often rapidly), increasing pathogenicity or virulence. Toxicants may degrade or accumulate in the environment and may change their hazardous properties through metabolic activation or deactivation within the organism (e.g., many carcinogenic chemicals) or through environmental biomethylation (e.g., mercury). These processes, however, are usually slower and much less substantial than the rapid changes typical of pathogenic organisms.

The toxicological paradigm presumes a direct passage from exposure to disease. With pathogens, points of feedback add additional complexity. For example, exposure to influenza may lead to development of immunity, clinical disease, or both. Toxicants can modulate these responses to infectious agents, as has been observed in gold miners exposed to both mercury and malaria (Silbergeld et al. 2005). Mercury affects host immune response, which can lead to increases in the incidence or clinical progression of malaria in a population (Gardner et al. 2009). We have incorporated these important feedbacks into our paradigm. As toxicology and infectious disease move toward integration, the state-of-the-art methods developed in understanding infectious disease ecology (e.g., using the basic reproductive number, *R*_0_, to gain insight into population-level infectious disease dynamics) should be considered.

## Incorporating Pathogens in the Toxicological Paradigm

### Potential barriers to integration in a common framework

#### Different meanings of the same terms

The disconnect between toxicology and infectious disease research is reinforced by inconsistencies in language and terminology. In toxicology, “exposure” is defined as external to the body; “dose” refers to the internalized level of a contaminant. Toxicokinetic processes occurring after internalization must be considered prior to impution or measurement of concentrations at sites of toxic action. In the language of infectious disease and immunology, “exposure” has different meanings. Nasal carriage of *Staphylococcus aureus* bacteria, for example, is often termed “exposure,” without distinction as to whether this is transient superficial contamination or a reflection of pathogen replication on an external body surface. Exposure in infectious disease also can refer to events after a foreign antigen is internalized and processed for presentation to the immune system, often measured serologically (e.g., through antibodies). The same biomarker—an antibody to a toxic agent (e.g., a hapten)—would be interpreted in toxicology as a measure of internal dose. In infectious disease, the concept of “exposure” is more general, hampering communication with toxicology and complicating ability to interpret the literature.

#### Lack of a common surveillance system

Environmental health surveillance systems focus on diseases related to the environment and on environmental exposures (International Epidemiological Association 2001; Thacker and Stroup 1996). Important environmental health surveillance programs include the Centers for Disease Control and Prevention (CDC)’s National Environmental Public Health Tracking Program (EPHT), National Biomonitoring Program (NBP), and National Health and Nutrition Examination Survey (NHANES). These programs differ: NBP collects data on biomarkers of exposure, NHANES collects information on both biomarkers of exposure and health status, and EPHT collects, analyzes, integrates, and disseminates data on exposures to environmental pollutants and potentially related health effects (CDC 2010). In contrast, infectious disease surveillance programs such as ProMED, FluView, and the WHO’s Global Outbreak Alert and Response Network are primarily response driven, based on detection of pathogens known to be associated with outbreaks or epidemics (CDC 2001, 2009; WHO 2009). Furthermore, the two fields differ in application of animal surveillance systems as sentinels for exposures or disease. In toxicology, animal sentinels have been used to detect specific hazardous conditions ever since canaries were carried into coal mines; more recent examples include tracking lead levels in wild rodents (Talmage and Walton 1991) or household dogs and cats (Berny et al. 1995). Infectious disease research incorporates animal sentinels more fully because of the close links between zoonotic pathogens and human health. For example, researchers track Hantaan virus using rats (Childs et al. 1985; Mills et al. 2009) and West Nile and influenza A viruses using wild birds (Liu et al. 2009; Munster and Fouchier 2009). Despite the potential utility of combining data from animal sentinels for integrated research, there is a lack of sufficient overlap and communication between the fields of animal and human disease research (Halliday et al. 2007; Rabinowitz et al. 2005). In general, common databases that combine information on both toxicant and pathogen exposures are lacking. There are examples where crossover between infectious disease and environmental health surveillance could increase capacity for interdisciplinary studies. For example, NHANES separately assesses exposures to environmental toxicants such as mercury (McDowell et al. 2004) and prevalence of hepatitis E virus seropositivity (Kuniholm et al. 2009), but NHANES has not been used to test interactions between pathogens and toxicant exposures.

#### Lack of defined methods for integrative risk assessment

Risk assessment is a key tool in environmental health sciences, using toxicology in determining risks to individuals or populations through the steps of hazard identification (determining if an agent potentially poses a threat if persons were to be exposed to it); exposure assessment (considering if and how people might come into contact with the hazardous agent); and dose–response assessment (determining relationships between the dose of the agent and adverse biological responses). Taken together, this information leads to the fourth step: characterizing the risk of the agent for given populations. The application of risk assessment methodologies to pathogens, although not straightforward, is evolving.

The emphasis on chemical toxicants at the U.S. EPA has facilitated the development of increasingly complex risk assessment methodologies. In comparison, assessment of pathogen risks has been applied primarily to food safety issues (Mota et al. 2009) but generally is much less developed largely because of the complexity of pathogen development and infectious disease transmission in the environment. The dynamic nature of microbial populations in terms of number and state, as discussed above, prevents direct application of toxicological models to infectious disease risk assessment (Ross and McMeekin 2003). Arguments against microbial risk assessment cite the need for extensive information about bacterial behavior under particular conditions, details of these conditions, and host responses in order to model risk (Toze et al. 2010). At present, the field of microbial risk assessment offers relatively few guidelines for incorporating data from multiple studies or at multiple levels of analysis, in contrast to recent guidelines for chemical risk assessment (National Research Council 2009). As a result, few microbial risk-assessment– based policies have been implemented. The lack of formal risk assessment methods for pathogens has posed considerable challenges to regulatory agencies, such as in the case of the Food and Drug Administration’s attempts to assess risks to human health associated with use of antimicrobials in animal feeds (Aarestrup et al. 2008).

Moreover, these differences in risk assessment can impede policy decisions that require a balancing of risks between microbial and chemical exposures, as evidenced in the trade-offs between waterborne arsenic and cholera in Bangladesh (Lokuge et al. 2004). Our integrated paradigm could help to advance research to address these barriers to integrated risk assessment.

#### Sample size concerns

In designing studies to evaluate pathogen–toxicant interactions, consideration of sample size is critical. A study sufficiently powered to detect the main effect from one agent may be underpowered to detect an interaction of similar magnitude between two agents (Brookes et al. 2004). Biostatistical literature on study design for subgroup interactions in clinical trials or for gene–environment interactions may prove relevant to designing the research we propose here. These works suggest increasing sample size 4‐fold to power a study to test interaction on the additive scale (Brookes et al. 2004) or altering sampling strategy to improve efficiency (Weinberg 2009).

### Unifying factors lending support for an integrated framework

#### Focus on upstream interventions

Important unifying factors support overcoming the barriers discussed above to integrate toxicology and infectious disease research. Fundamentally, both fields promote prevention of exposure as the most efficient way to reduce disease risks. In environmental health, this focus on preventing exposure is exemplified in the “hierarchy of controls,” in which interventions at the source of exposure are preferred to reduce or eliminate human exposure. In contrast, infectious disease control efforts largely focus on receptors, through achieving herd immunity by reducing the pool of susceptible individuals through quarantine or vaccination. Some interventions before actual infection, such as metaphylaxis (treatment in the absence of disease), can have negative consequences, such as driving selection of drug resistance within the pathogen community (Aarestrup et al. 2000). Some of the most effective opportunities for primary prevention in infectious disease, prior to exposure of a human or animal host, often involve environmental interventions such as elimination of vector habitats and protection of food or water sources. This shared focus on the value of upstream interventions is an important driver for integrating infectious disease into environmental health.

#### Spatial context

Researchers in environmental health have developed and employed methods for the study of spatially distributed risks, including cluster analysis and small-area studies that can detect geographically definable areas of elevated exposures or disease in certain populations. These approaches rely on the assumption that proximity to a toxicant source is a risk factor for exposure. For example, studies using spatial techniques have found associations between distance from highways (as a proxy for exposure to air pollutants) and symptoms of respiratory distress (Boothe and Shendell 2008; Braback and Forsberg 2009). The same tools have proven useful in studying infectious diseases influenced by spatial or landscape factors (Kitron 1998), as well as identifying risk factors for disease spread (Eisen et al. 2007; Glass et al. 1995). Considering its utility for both environmental health and infectious disease studies, spatial modeling has the potential to be a cornerstone in the development of integrated research.

#### Quantitative models of exposure

Human exposure modeling that incorporates all potential routes of exposure is a central aspect of risk assessment. Quantitative models are used to estimate toxicant exposure at the ambient or personal level and are valuable when local-scale measurements are inappropriate, unfeasible, or cost-prohibitive. For example, food consumption data enable estimation of pesticides consumed or probability of exposure to foodborne pathogens under given conditions. Modeling is applied in infectious disease research for estimating disease transmission within a population and for comparing various public health interventions. These models typically estimate population-level changes and transitions through defined disease states using differential equations. Like spatial modeling, this shared approach provides an opportunity for increased collaboration and communication.

#### Biomarkers and omics

Biomarkers are another unifying factor between these fields. Biomarkers are measurable molecular changes in the body (e.g., blood, tissue) that provide information on three main characteristics: exposure, susceptibility, and disease (National Research Council Committee on Biological Markers 1987). They are used as indicators of events along the toxicological pathway, including toxicokinetics (exposure, internal dose, and biologically effective dose) and toxicodynamics (early biological effect, altered structure and function, and clinical disease) (National Research Council Committee on Biological Markers 1987). Infectious disease research has long used biomarkers through the direct measurement of pathogens in body compartments or excreta and more recently through serological analysis of antibodies to pathogens. Biomarkers also can be used to distinguish between different genetic strains of pathogens, which can help to identify the source or transmission pathway of a disease. Our proposed integrated paradigm incorporates both infectious agents and toxicants as potential exposures on the pathway to disease.

New technologies empowered by genome projects have generated considerable attention in both toxicology and infectious disease. Through the National Center for Toxicogenomics at the NIEHS, which has the goal of identifying genetic polymorphisms that modulate response pathways, the field of toxicology has adopted a systematic approach to omics that is relevant to pathogens as well. These technologies have produced valuable biomarkers of exposure and early biological responses in clinical studies (Beger et al. 2010). However, the predictive power of many biomarkers is largely unvalidated, potentially producing misleading information (Nilsson et al. 2009). Moreover, in the absence of more traditional information on the context of toxicant exposures, omics biomarkers may be difficult to interpret for retrospective exposure assessment (Scheepers 2008). These omics technologies promise to deliver a molecular “footprint” for risk factors that could be identified and traced through the steps of the traditional toxicological paradigm (Guyton et al. 2009; Ramos et al. 2007; Schnackenberg and Beger 2006; Wetmore and Merrick 2004). Attempts to produce this “holy grail” include relatively simple analyses of base-pair substitutions (Lasky and Silbergeld 1996) and collection of dense data sets derived from microarray gene expression data and proteomics (Frijters et al. 2007). To a large extent, these applications have used cross-sectional measurements, which may explain why surprisingly little value has been extracted in terms of explaining dynamic events between exposure and outcome. A recently published analysis of microarray gene expression data for six carcinogens (of both chemical and pathogen origin) demonstrates the importance of measuring these signals over time in order to understand dynamic events between exposure and outcome (Mathijs et al. 2009). In contrast, the use of omics methods in infectious disease research has been extensive and valuable in contributing to understanding events from exposure source to disease. Pathogen “exposure” often is defined very precisely by genotype, a level of analysis that provides important information on agent source and networks of host–host transmission. For example, our understanding of methicillin-resistant *Staphylococcus aureus* (MRSA) dissemination in humans and animals depends on the use of genotypic methods such as multilocus sequence testing to identify outbreaks of disease (Kock et al. 2009) and to detect otherwise unrecognized disease clusters (Mellmann et al. 2006). Whole-genome methods have revealed early evolutionary changes in the H1N1 influenza A virus (Ramakrishnan et al. 2009; Vincent et al. 2009) and differential virulence genes in porcine enteropathic *Escherichia coli* (Bruant et al. 2009). Genetic analysis of *Campylobacter jejuni* bacteria has provided insight into observed associations between *C. jejuni* infection and autoimmune peripheral neuropathies (Dingle et al. 2001).

## Examples of Research Exemplifying the Integrated Paradigm

### Pathogen–toxicant interactions

Several chronic diseases have been strongly associated with toxic agents. Pathogens can also cause some of these same diseases, as well as interact with toxicants in the etiology of diseases that are typically considered to be toxicant associated. For example, NIEHS research and funding highlight cancer as a disease associated with gene–toxicant interactions. However, pathogens, particularly viruses, are increasingly being recognized as carcinogens (Fontham 2009; Schulz 2009).

A century ago, Rous (1910) provided evidence of a role for viruses in cancer, but dogma during the next several decades was dominated by resistance to this possibility (Epstein 1971). More than 70 years ago, Rous and colleagues (Rogers and Rous 1951; Rous and Kidd 1936, 1938) also presented evidence that viruses and chemical toxicants can interact in carcinogenesis. The Shope papilloma virus causes warts or papillomas on rabbits’ skin (Shope and Hurst 1933); in some animals these papillomas were observed to progress to malignant carcinomas after several months (Rous and Beard 1935). Meanwhile, coal tar applied to rabbits’ skin was observed to result in warts, which occasionally became malignant after longer time periods (Rous and Kidd 1938). In a combined experiment of coal tar application to rabbit skin followed by injection of Shope papilloma virus, Rous and Kidd (1938) observed that carcinomas developed more rapidly than under either exposure alone, indicating an interaction between chemical and viral carcinogens.

Despite this early evidence that papilloma viruses can interact with chemicals in carcinogenesis in animals, little research on pathogen–chemical interactions in cancer has been performed. Assessment of potential interactions of chemicals with HPV was limited by delayed recognition of HPV as a carcinogen. In the late 1980s Layde and Broste (1989) realized that the potential role of smoking in cervical cancer etiology could not be appropriately assessed without recognition of the relevant infectious agent as a potential confounder or effect modifier. Now that HPV has been established as the primary risk factor for cervical cancer, epidemiological methods for studies of potential chemical cofactors such as smoking, oral contraceptive use, and diet have improved, and remaining methodological challenges have been identified (Castellsagué and Muñoz 2003; Castellsagué et al. 2002). Recent studies that have assessed the risk of smoking while accounting for HPV status provide evidence that smoking may act as a cofactor in cervical cancer (Castellsagué et al. 2002; Castellsagué and Muñoz 2003). Several potential biological mechanisms have been proposed for this interaction, including nicotine causing DNA damage in the cervix and chemical modulation of immune response to HPV (Castellsagué et al. 2002; Gunnell et al. 2006).

Evidence is emerging for a potential role of infectious agents in other chronic diseases. Bidirectional interactions have been observed for mercury and pathogens. Mercury can alter the immune response to coxsackie B3 virus (CB3V) (Ilbäck et al. 1996), with resulting increases in postinfection pathology, including autoimmunity (Nyland et al. 2004; South et al. 2001). Also, infection by CB3V alters the toxicokinetics of mercury, resulting in increased levels of mercury in heart tissue (Frisk et al. 2008). These examples indicate that research on the etiology of chronic disease can benefit from an interdisciplinary approach that investigates both infectious agents and environmental toxicants and their potential interactions.

Limited research suggests interactions between toxicants and severity of infectious disease or acquisition of resistance to infectious diseases. Studies of coexposures to mercury and malaria (in both animal models and human populations) suggest that mercury may inhibit acquisition of immunity to malaria (Crompton et al. 2002; Silbergeld et al. 2005), but more research is needed on this and related topics.

### Key example: toxicant and viral interactions in liver cancer etiology

Research progress in the area of hepatocellular carcinoma (liver cancer) is a key example of research that successfully applies the toxicological model and other tools, such as biomarkers and genomics, to studying both environmental toxicants and pathogens in disease etiology (Groopman et al. 2005). This work helped motivate our research; it illustrates how our integrated paradigm can be applied to explore interaction between toxicants and infectious disease in an environmental context.

Early epidemiological studies indicated that aflatoxin, a fungal toxin that is a contaminant of food, was associated with increased risk of liver cancer (Groopman et al. 2005). However, these studies did not account for the potential role of hepatitis viruses (Groopman et al. 2005). Separate studies were conducted on HBV without assessing aflatoxin exposure. In 1981, results from a prospective cohort study of 22,707 men in China indicated that those men who tested positive for hepatitis B surface antigen were 223 times more likely to develop liver cancer than those men who tested negative (Beasley et al. 1981). The assessment of aflatoxin and hepatitis exposures separately did not account for the possibility that these two agents might interact with each other. The first investigations of coexposures to aflatoxin and HBV used biomarkers of exposure to both risk factors in a case–control study nested within a cohort of 18,244 people in Shanghai, China (Qian et al. 1994; Ross et al. 1992). Although aflatoxin and HBV each were independently associated with liver cancer, a dramatic synergistic interaction was observed between aflatoxin exposure and HBV infection (Table 1) (Qian et al. 1994), which has been replicated in additional studies (Wild and Montesano 2009). Several mechanisms may contribute to development of liver cancer; genetic factors, exposures to other toxicants such as alcohol, and other forms of hepatitis may play a role (Cha and Dematteo 2005). The potential role of genetic susceptibility factors of the host, as well as pathogen genetic factors (e.g., mutations in the HBV genome), highlights the importance of incorporating the newer techniques of genomics into cross-disciplinary research (Groopman et al. 2005).

**Figure 3:**
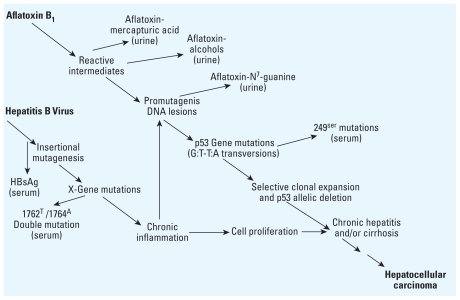
Potential molecular mechanisms of interaction between HBV and aflatoxin in the development of hepatocellular carcinoma, as elucidated by several biomarkers. HBsAg, hepatitis B surface antigen. Reprinted from Groopman et al. (2005) [Aflatoxin and hepatitis B virus biomarkers: a paradigm for complex environmental exposures and cancer risk. *Cancer Biomarkers* 1:5–14]; copyright (2005) with permission from IOS Press.

Figure 3 shows the molecular mechanisms by which HBV and aflatoxin may act both independently and synergistically in liver cancer etiology (Groopman et al. 2005). Although these mechanisms are not completely understood, they have been characterized through the use of biomarkers (Cha and Dematteo 2005; Groopman et al. 2005; Wild and Montesano 2009). This integrated biomarker research has led to the identification of potential strategies for liver cancer prevention that address the role of either the viral or toxicant exposures or both (Groopman et al. 2005; Wild and Montesano 2009). These strategies include HBV vaccination, reduction of aflatoxin exposure, and, notably, chemoprevention by altering aflatoxin metabolism, which may be important in those populations where primary prevention is currently difficult (Groopman et al. 2005). Biomarkers have been used as intermediate end points to optimize the evaluation of these agents in trials (Groopman et al. 2005).

## Colon Cancer: Potential Research Following the Integrated Paradigm

As with liver cancer, colon cancer research might benefit from interdisciplinary investigation of potential microbial–chemical interactions. The potential for such interactions in colon cancer was hypothesized by Weisburger in 1971 but not directly tested. Weisburger (1971) speculated about several possible mechanisms of colon carcinogenesis involving diet, activity of gut flora, or both. As one possibility, he hypothesized that bacterial action might release active intermediates from ingested chemicals such as aromatic amines (Weisburger 1971). Recent research indicates that both microbes and chemicals may be risk factors for colon cancer, but these have been explored as separate entities. Epidemiological studies, including studies using DNA adducts as biomarkers, indicate that exposure to heterocyclic amines (HCAs) and polycyclic aromatic hydrocarbons (PAHs) found in well-done meat may be associated with risk of colon cancer via DNA damage (Ramesh et al. 2004; Rohrmann et al. 2009; Sinha et al. 2005). Genetic risk factors, such as polymorphisms of the UDP-glucuronosyltransferase 1A7 gene, may interact with these dietary chemicals in colon cancer etiology (Butler et al. 2005). On a separate track, recent microbiology research indicates that enterotoxigenic *Bacteroides fragilis* bacteria may contribute to colitis (Rabizadeh et al. 2007) and to the development of colon tumors via T_H_17-mediated inflammation (Wu et al. 2009). Thus, bacterial-associated inflammation of the colon (colitis) may contribute to colon cancer, as viral-associated inflammation of the liver (hepatitis) contributes to liver cancer. HCAs and PAHs may exhibit chemical carcinogenicity in the colon, as aflatoxin exhibits liver carcinogenicity. Given the toxicant–pathogen synergy in liver cancer, we hypothesize that similar interactions may occur in colon cancer etiology. To test this hypothesis, a study could explore potential interactions between ETBF (enterotoxigenic *Bacteroides fragilis*)bacteria and HCAs or PAHs. Our integrated paradigm provides a common framework for collaborators to explore such interdisciplinary research questions.

## Recommendations for Future Funding and Research

Research initiatives should be designed to foster collaborations among researchers in infectious disease and environmental health, specifically toxicology. Large, population-based studies should include sample collections that enable assessment of exposures to environmental agents as well as to pathogens. In experimental research, the assessment of immunotoxicity in animal models should go beyond characterization of immune parameters (i.e., cell subsets, cytokine production, cell surface markers) and standard measures of function (i.e., sheep red blood cell response, lipopolysaccharide stimulation) to models that incorporate analysis of response to infectious agents.

In concert with the strategic plan of the NIEHS, collaboration with other agencies within the National Institutes of Health should be promoted to stimulate cross- disciplinary research (NIEHS 2006). A high priority should be given to cross-disciplinary research on those diseases for which toxicological and pathogen etiologies have separately been identified. This may be accomplished best through the issuance of joint requests for proposals and the creation of specific study sections.

The integrated paradigm presented in this review could guide education and training of environmental health researchers and practitioners. Improving interdisciplinary understanding is critical—and environmental health professionals should fully engage in dialogue, research, and policy development in these areas. To achieve this, formal training in integrated research designs must be brought more overtly into environmental health curricula. Improving communication among researchers is instrumental in building and maintaining bridges across separated fields. Conferences jointly sponsored by core government and professional organizations in environmental health and infectious disease can foster networking and collaboration, along with grant programs and other funding. This provides a venue for the sharing of research to spark ideas for studies to investigate possible interactions. Successful models, such as EID, offer opportunities for expansion.

Integrating surveillance systems—of both animals and humans—is critically important, and improving cross-utilization and communication among existing surveillance programs is a first step. Collaborative efforts will be needed to design these systems for applicability in both infectious disease and toxicology. Databases of animal and human health surveillance data should be linked, and barriers of incompatible forms of data collection and management should be eliminated (Scotch et al. 2009). Increased support for CDC biomonitoring and surveillance programs (NHANES, NBP, EPHT) should be allocated to ensure funds for sample collection and analysis of both chemicals and pathogens.

## Conclusions

The toxicological paradigm has not been engaged previously as a mechanism for understanding the relationship between environmental health and infectious diseases. Recognizing and incorporating the common tools of spatial analysis, risk assessment, exposure modeling, biomarker analysis, and genomics will enhance a comprehensive approach to studying the role of both toxicants and pathogens in chronic disease etiology. Reframing the toxicological paradigm provides a roadmap for interdisciplinary research and a guide for research conducted at this important interface, and may help public health practitioners to weigh the risks posed by the constant barrage of environmental exposures on the human system and determine how to best mitigate them. Funding agencies, academic departments of environmental health, and researchers in related fields need to work together to broaden the scope of environmental health and to drive innovative research.

In a broader sense, the limits of the current focus of environmental health on toxicology do not apply only to the exclusion of pathogens. Classic reductionist thinking in toxicology focuses on “one toxicant, one outcome” research. This neglects not only infections but also other comorbidities that burden much of the U.S. and worldwide populations, such as obesity and diabetes. With most of the U.S. population taking drugs daily to treat these comorbidities, research must account for the realities of complex exposure scenarios. If basic research is to increase our ability to predict the consequences of exposure to environmental chemicals, we must embrace nonreductionist thinking and design experimental models that emulate human experience, including but not limited to the interactions between infections and toxicological exposures.

## Figures and Tables

**Table 1:** Combined effects of HBsAg positivity and presence of urinary aflatoxin biomarkers^a^ on risk of hepatocellular carcinoma in Shanghai.

	Aflatoxin negative			Aflatoxin positive		
HBsAg	Cases	Controls	RR^b^ (95% CI)	Cases	Controls	RR^b^ (95% CI)
Negative	5	134	1.0	13	102	3.4 (1.1–10.0)
Positive	9	24	7.3 (2.2–24.4)	23	7	59.4 (16.6–212.0)

Abbreviations: CI, confidence interval; HBsAg, hepatitis B surface antigen. Reprinted from Qian et al. (1994) with permission from the American Association for Cancer Research.

a AFB_1_, AFP_1_, AFM_1_, and AFB_1_-N^7^-guanine.

b Adjusted for cigarette smoking.
